# Pattern and risk factors for childhood injuries in Dar es Salaam, Tanzania

**DOI:** 10.4314/ahs.v21i2.42

**Published:** 2021-06

**Authors:** Robert Moshiro, Francis F Furia, Augustine Massawe, Elia John Mmbaga

**Affiliations:** 1 Department of Paediatrics, Muhimbili National Hospital, Dar es Salaam, Tanzania; 2 Department of Paediatrics and Child Health, School of Medicine, Muhimbili University of Health and Allied Sciences, Dar es Salaam, Tanzania; 3 Department of Epidemiology and Biostatistics, School of Public Health and Social Sciences, Muhimbili University of Health and Allied Sciences, Dar es Salaam, Tanzania

**Keywords:** Childhood injuries, risk factors, Dar es Salaam, Tanzania

## Abstract

**Background:**

Injuries contribute to morbidity and mortality in children. This study was carried out to describe the pattern of childhood injuries and associated risk factors in Dar es Salaam, Tanzania.

**Methods:**

This case control study was conducted in six selected health facilities in Dar es Salaam, Tanzania. Data were collected using a structured questionnaire. Cases and controls were children below 18 years who had suffered injuries and those without injury associated condition respectively.

**Results:**

A total of 492 cases and 492 controls were included in the study, falls (32%), burns (26%), Road Traffic Injuries (14%) and cuts (10%) were the major types of injuries identified. Younger parents/guardians {Adjusted odds ratio (AOR)= 1.4; 95% CI: 1.4 -3.6}, more than six people in the same house (AOR= 1.8; 95% CI: 1.3–2.6), more than three children in the house {AOR= 1.4; 95% CI (1.0–2.0)}, absence of parent/guardian at time of injury occurrence (AOR= 1.6; 95% CI: 1.1–2.3), middle socio-economic (AOR=1.6; 95%CI: 1.1–2.4) and low socio-economic status (AOR= 1.5; 95% CI: 1.0–2.1) were independent risk factors for childhood injury.

**Conclusion:**

Falls, burns and road traffic injuries were the main injury types in this study. Inadequate supervision, overcrowding, lower socio-economic status and low maternal age were significant risk factors for childhood injuries.

## Background

Injuries are damages to human body following exposure to mechanical, chemical, thermal or radiation energy. They contribute to significantly high morbidity and mortality and it is estimated that about 35 million deaths globally were attributed to unintentional injuries between 2007 and 2017.^1^ Children are among the most affected population with injuries and in 2015 about 177,000 children were estimated to have died of injuries in Africa.^2^–^4^

Unintentional childhood injuries include falls, road traffic injury and burns contribute to high mortality and morbidity with Africa, predominantly male and younger children are more affected.^5^, ^6^ Intentional injuries also contribute to severe morbidity and long-term disability in children.^7^

Several factors contribute to high burden of childhood injuries in lower and income countries; these include behavior of children, employment of parents, social supports, rapid urbanization and motorization with increased use of motorcycles.^8^–^11^ Understanding the pattern and factors contributing to injuries is key to designing and implementing preventive measures, therefore this study was aimed at describing the pattern of injuries and contributing factors among children in Dar es Salaam, Tanzania.

## Methodology

### Study Design and Setting

This matched case control study was conducted between August 2011 and March 2012 in six health facilities in Dar es Salaam city which has an estimated population of 5 million inhabitants 40% of which are children.12 Participants were recruited in three regional hospitals (Mwananyamala, Amana and Temeke) and three health centres (Buguruni, Mbagala and Sinza).

### Study population

Children below 18 years who presented to the facilities were eligible, cases were recruited in the casualty departments while controls were recruited in recruited in the follow up and child health clinics in the same facility. Injury was defined as physical damage to the body resulting from exposure to mechanical, thermal, electrical or chemical energy. Birth injuries were excluded from this study.

### Sample Size and Power

Werneck et al report from a study conducted in Brazil to assess the effect of parity on burn injury was used for calculating sample size (Werneck and Reichenheim 1997).13 Using Fleiss formula, a minimum 265 pairs of cases and control were needed to give a power of 90% with 95% confidence interval, using case; control ratio of 1:1 and detection odds ratio of 2.14

### Data collection

Two research assistants were placed at each facility. . An attending doctor on duty together with research assistant identified children with injuries and collected data from accompanying parents/guardians using structured questionnaires. Cases were recruited fromthe outpatient casualty department. After identifying the case, age and sex matched control was then identified the same day in the same facility. Controls aged below 5 years were recruited from reproductive and child health follow up clinic, which offers routine immunization and growth monitoring for under-five children. Controls aged 5 years and above were recruited from children who visited outpatient clinic due to other illnesses apart from injury. If a matched control was not found on the same day that case was recruited, recruitment was made on the following day. Recruitment of participants and data collection process was stopped when sample size was achieved.

Information collected included nature of injury as the main variable, other information included demographics; age, sex, marital status, and occupation, level of education, number of children in the family and number of occupants in the household. Source of household energy and/or light and presence of chemicals within reach of children was also inquired.

### Data analysis

Data were entered into Statistical Package for Social Sciences (SPSS) version 15 for analysis. Data were summarized into two-way table and were analyzed using Chi Square test and Student t test to determine differences between categorical and continuous variables respectively. Logistic regression was used to estimate Odds Ratios (ORs), Adjusted Odds Ratio (AOR) and 95% confidence intervals to ascertain independent risk factors for injury. Risk factors with a p value less or equal to 0.2 were selected for multivariate logistic regression model. All statistical tests were two-tailed and significance level set at 5%.

Socio-economic status was classified into poor, middle and wealthier categories. These scales were generated based on analysis of items such as TV, radio and house characteristics, type of toilet, and types of roofing material. The scale had a Cronbach's alpha of 0.823 as a measure of internal consistency. Logistic regression was used to estimate Odds Ratios (ORs), Adjusted Odds Ratio (AOR) and 95% confidence intervals to ascertain independent risk factors for injury. Risk factors with a p value less or equal to 0.2 were selected for multivariate logistic regression model. All statistical tests were twotailed and significance level set at 5%.

### Ethical consideration

Muhimbili University of Health and Allied Sciences (MUHAS) Institution Review Board granted ethical approval for this study, and permission to conduct this study was granted by involved hospital administrations. Before recruitment informed consent was sought from parents/guardians and assent was obtained from children aged ≥10 years and above.

## Results

### Social-demographic characteristics of the study participants

A total of 492 cases and 492 controls were recruited from six selected facilities in the city. The mean age for cases and controls were 55.2 ± 4.5 and 54.8 ± 4.5 months, respectively. Male children accounted for 58% of injured children. The age group 1–4 years accounted for 61% of all injured children. Parents/ guardians of children who presented with injuries (cases) were significantly younger than those of controls. Socio-economic status of parents in the control group was noted to be higher than in the cases, p = 0.001 (Table 1).

**Table 1:** Comparisons of socio-demographic characteristics of parents/guardian among the cases and controls

Variable	Total	Cases n(%)	Control n(%)	P value
**Maternal age** **(yrs)**				
15 – 24	237	119 (24.2)	118 (24.0)	0.005
25 – 34	505	273 (55.5)	232 (47.2)	
35+	242	100 (20.3)	142 (28.9)	
Total	984	492	492	
**Education**				
No education	83	38 (7.7)	45 (9.1)	0.5
Primary	567	291 (59.1)	276 (56.1)	
Secondary+	334	163 (33.2)	171 (34.8)	
Total	984	492	492	
**Marital status**				
Single	163	73 (14.9)	90 (18.3)	0.20
Married	713	369 (75)	344 (69.9)	
Separated	108	50 (10.2)	58 (11.8)	
Total	984	492	492	
**Occupation**				
Peasant	45	30 (6.1)	15 (3.0)	0.06
Employed	232	121 (24.6)	111 (22.6)	
Petty trader	390	186 (37.8)	204 (41.5)	
Housewife	293	147 (29.9)	146 (29.7)	
Others	24	8 (1.6)	16 (3.2)	
**SES**				
Poor	318	176 (37.5)	142 (29.4)	0.001
Middle	319	163 (34.7)	156 (32.2)	
Wealthier	316	130 (27.8)	186 (38.4)	

As described in Table 2, children with injuries were more likely to come from households with three or more children (p<0.001), and those with 6 or more people (p=0.001) compared to controls. Children with injuries were more likely to be left alone at home as compared to controls (p=0.001), and more children with injuries as compared to controls were left with minor caretakers, p = 0.001.

**Table 2:** Comparisons of socio-demographic characteristics of children among the cases and controls

Variable	Category	Total	Cases n(%)	Control n(%)	P value
Age (yrs)	<1	35	16 (3.2)	19 (3.8)	0.9
	1 – 4	601	301 (61.2)	300 (61.0)	
	5 – 9	223	114 (23.2)	109 (22.2)	
	10 – 17	125	61 (12.4)	64 (13.0)	
	Total	984	492	492	
Sex	Male	568	285 (57.9)	283 (57.5)	0.9
	Female	416	207 (42.1)	209 (42.5)	
	Total	984	492	492	
No of children	1 -2	675	304 (62.0)	371 (76.0)	<0.001
in the house	3+	306	186 (38.0)	120 (24.0)	
	Total	981	490	491	
Number of	3 – 5	679	307 (62.4)	372 (75.6)	0.001
People in house	6 +	305	185 (37.6)	120 (24.4)	
	Total	984	492	492	
Child left home	Yes	311	192 (39.6)	119 (24.2)	0.001
Alone	No	665	293 (60.4)	372 (75.8)	
	Total	976	485^†^	491	
Child left with	Yes	453	270 (55.7)	183 (37.2)	0.001
a minor caretaker	No	524	215 (44.3)	309 (62.8)	
	Total	977	485^†^	492	

### Pattern of Injuries

**Figure 1:**
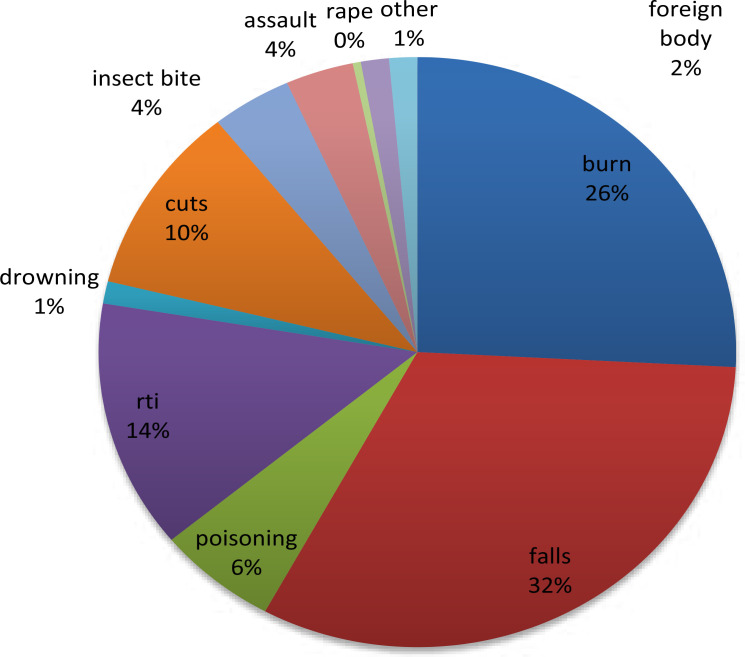
Pattern of injuries among cases

Injuries noted in this s tudy included fall (32.1%), burns (25.8%), RTI (13.6%) and cuts (10.4%). Seventeen children (3.5%) reported to have been assaulted mostly by peers. Seven (2%) children had swallowed a foreign body, two female children (0.4%) were sexually assaulted (raped) and six children (1.2%) had drowned. (Figure 1).

Majority of falls occurred at home (81%) affecting mostly children aged 1–4 years (68.4%). Burns occurred predominantly at home (98.4%) and were caused by hot foods (44.1%) and water (40.9%). Majority (80.7%) of children who sustained road traffic injuries were pedestrians, and these injuries resulted mainly from motorcycles (53.7%) and cars (38.8%). Kerosene (46.7%) and medicines (43.3%) were the predominant types of poisons reported.

**Figure 2:**
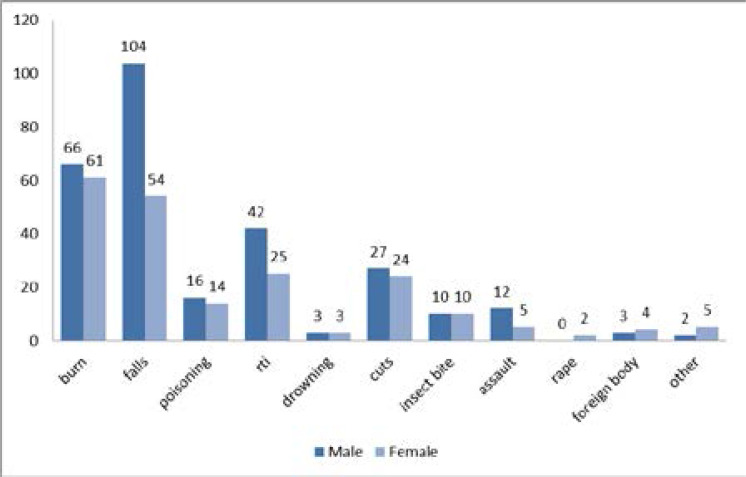
Number of male and female cases recruited

Figure 2 describes type of injury according to sex of children; more male children were injured (58%) as compared to female children (42%). Most injuries in this study (70.0%) were reported to have occurred at home while 7.1% and 9.4% occurred at school and sport areas/venues, respectively.

### Risk Factors for Childhood Injuries

Child related independent risk factors for injuries are living child unsupervised at home and increased number of children and adults in the household as described in Table 3. Children left at home alone were about two times more likely to be injured as compared to those with proper supervision after adjusting for confounders (AOR = 1.6, 95% CI (1.1; 2.4). Similarly, children left with minor caretakers had 1.5fold increased odds of injury (AOR = 1.6, 95% CI:11–2.3).

**Table 3:** Logistic regression of all independent risk factors for injuries among children aged less than 18 years in Dar es Salaam

Variable	Category	OR (95% CI)	*AOR (95% CI)	xp – value
Child	<1	1	1	
age (years)	1 – 4	1.2 (0.6–2.3)	1.2 (0.5–2.9)	0.67
	5 – 9	1.2 (0.6–2.5)	1.1 (0.7–1.9)	0.54
	10 – 17	1.1 (0.5–2.4)	1.0 (0.5–1.6)	0.9
Sex	Male	1	1	
	Female	1.0 (0.8–1.3)	1.0 (0.8–1.3)	0.99
Child left	No	1	1	
alone	Yes	2.0 (1.5–2.7)	1.6 (1.1–2.3)	0.02
Child left	No	1	1	
with a minor	Yes	2.1 (1.6–2.7)	1.5 (1.1–2.2)	0.010
caretaker				
Number of	1 – 2	1	1	
children in	3+	1.7 (1.3–2.2)	1.4 (1.0–2.0)	0.032
the household				
Number of	3 – 5	1	1	
people in the	6+	1.8 (1.4–2.4)	1.8 (1.3–2.6)	0.001
household				
Maternal age	35+	1	1	
	15–24	1.4 (0.9–2.0)	2.2 (1.4–3.6)	0.001
	25–34	1.6 (1.2–2.2)	2.0 (1.4–2.9)	<0.001
Occupation	Peasant	1	1	
	Employed	0.5 (0.2–1.0)	0.9 (0.4–2.0)	0.91
	Petty	0.4 (0.2–0.8)	0.6 (0.3–1.2)	0.18
	trader	0.5 (0.2–0.9)	0.6 (0.3–1.2)	0.22
	Housewife			
Marital status	Single	1	1	
	Married	1.3 (0.9–1.8)	1.3 (0.9–1.9)	0.20
	Separated	1.0 (0.6–1.7)	1.0 (0.6–1.8)	0.93
Economic	Wealthier	1	1	
status	Middle	1.5 (1.1–2.0)	1.6 (1.1–2.4)	0.011
	Poor	1.7 (1.3–2.4)	1.5 (1.0–2.1)	0.036

OR-Odds ratio; AOR- Adjusted odds ratio; CI- Confidence Interval

x p-value for adjusted odds ratio

* All variables are adjusted for others in the table

Living in households with three or more children had 1.7 times increased probability of injury compared to those with 1–3 children (AOR=1.3, 95% CI:1.0 – 1.9)}. Moreover, households with more than 6 individuals had almost twice the likelihood of childhood injury as compared to those households with less than 6 people {AOR=1.7, 95%CI: 1.3–2.3).

Maternal age and socio-economic status, number of people in the household and leaving children alone or under supervision of minors were noted to be independent predictors of childhood injuries as described in Table 3. Young mothers had 2 fold increased risk of childhood injuries as compared to older mothers. Households with poor and middle socio-economic level had about 1.5 fold increased likelihood of childhood injury as compared to wealthier ones.

## Discussion

This case control study was carried out to determine pattern and risk factors for childhood injuries in Dar es Salaam, Tanzania. Falls, burns, RTIs and cuts were the main types of injury reported. Overall, cases were more likely to be males than females in this population. Risk factors for injuries noted in this study included poor children supervision, overcrowding, having more than 3 children in the house, lower economic status and low maternal age.

Male children were predominantly injured in this study; this is consistent with other reports and is usually attributed to higher level of activities as compared to female children.^15^,^16^ Younger children (1–4 years) are usually very curious in exploring their immediate environment thereby exposing themselves to hazards, were more involved in injuries in this study.^17^

Common childhood injuries reported indel from sub-Saharan Africa were also noted in this study, which include falls, burn, RTI, poisoning and drowning.^18^–^22^ Majority of injuries was sustained at home like reports from previous studies, raising serious concerns as homes are expected to be safe.^16^,^17^ There is a need increase advocacy and promote strategies for making homes safe.

Younger children were predominantly injured through falls, although majority of falls result in mild injuries, they have potential for causing serious morbidities including brain injuries contributing to mortality.^16^, ^23^ Scalding was also commonly reported among younger children (below 2 years), these occurred at home and could have happened in the presence of adults, as has been the case from other studies.^9^,^16^ Burn injuries result in serious deformity and contribute to high mortality among children.^24^ Observed findings in this study calls for effort to advocate for anticipatory guidance and supervision of young children to prevent injuries from fire and hot liquids/foods.

The surge of motor traffic injuries in many urbanized African regions is attributed to changes in public transportation following introduction of motorcycles, in this study motorcycle related RTI were common among pre-school and school children. This finding, which is similar to other findings from the region, underscores the need for improved infrastructure and enforcement of traffic laws in Tanzania.^8^, ^25^

Poisoning involving medicines and household items like kerosene is affect young children, in this study poisoning was also reported contributing to injury consistent with other reports from other studies in the region.^6^, ^26^,^27^. Therefore there is a need to keep emphasis of storing medicine and hazardous household items out of children's reach.

Childhood injuries are common in low- and middle-income countries and are influenced by several factors including family income, maternal education, family structure and issues related to accommodation.^21^,^29^ Improved quality of life and parenting education are important especially at community level. Proper planning in big cities like Dar es Salaam, which are rapidly growing and over-populated, should be considered to make them safe cities for children.^11^

Children supervision in homes plays an important role in prevention of childhood injuries, therefore efforts for promoting improved childhood supervision and safe household environment should be emphasized..^16^ Similarly the habit of leaving older children to supervise younger ones should be strongly discouraged.

This study was conducted in the hospital setting, therefore recruited participants are those with moderate to severe injuries, minor injuries which are the most common occurring in the community might not be presented to health facilities, therefore the findings of this study may not be generalizable to the community setting. Another important limitation of this study was not grading injuries and following up of injured participants to determine what treatment was offered and their outcomes making it difficult to ascertain the consequence of injuries.

## Conclusion

Falls, burns and road traffic accidents were the predominant injuries. Injuries were more common among unsupervised children and those from poor and overcrowded households. There is a need for initiating community campaigns to raise awareness on childhood injuries focusing on making home environment safe and improving supervision for children.
